# Mechanisms of inflammatory responses and development of insulin resistance: how are they interlinked?

**DOI:** 10.1186/s12929-016-0303-y

**Published:** 2016-12-03

**Authors:** Kanwal Rehman, Muhammad Sajid Hamid Akash

**Affiliations:** 1 Institute of Pharmacy, Physiology and Pharmacology, University of Agriculture, Faisalabad, Pakistan; 2 Department of Pharmaceutical Chemistry, Government College University, Faisalabad, Pakistan

**Keywords:** Insulin resistance, Insulin sensitivity, Pro-inflammatory mediators, Transcriptional pathways, Diabetes mellitus

## Abstract

**Background:**

Insulin resistance (IR) is one of the major hallmark for pathogenesis and etiology of type 2 diabetes mellitus (T2DM). IR is directly interlinked with various inflammatory responses which play crucial role in the development of IR. Inflammatory responses play a crucial role in the pathogenesis and development of IR which is one of the main causative factor for the etiology of T2DM.

**Methods:**

A comprehensive online English literature was searched using various electronic search databases. Different search terms for pathogenesis of IR, role of various inflammatory responses were used and an advanced search was conducted by combining all the search fields in abstracts, keywords, and titles.

**Results:**

We summarized the data from the searched articles and found that inflammatory responses activate the production of various pro-inflammatory mediators notably cytokines, chemokines and adipocytokines through the involvement of various transcriptional mediated molecular pathways, oxidative and metabolic stress. Overnutrition is one of the major causative factor that contributes to induce the state of low-grade inflammation due to which accumulation of elevated levels of glucose and/or lipids in blood stream occur that leads to the activation of various transcriptional mediated molecular and metabolic pathways. This results in the induction of various pro-inflammatory mediators that are decisively involved to provoke the pathogenesis of tissue-specific IR by interfering with insulin signaling pathways. Once IR is developed, it increases oxidative stress in β-cells of pancreatic islets and peripheral tissues which impairs insulin secretion, and insulin sensitivity in β-cells of pancreatic islets and peripheral tissues, respectively. Moreover, we also summarized the data regarding various treatment strategies of inflammatory responses-induced IR.

**Conclusions:**

In this article, we have briefly described that how pro-inflammatory mediators, oxidative stress, transcriptional mediated molecular and metabolic pathways are involved in the pathogenesis of tissues-specific IR. Moreover, based on recent investigations, we have also described that to counterfeit these inflammatory responses is one of the best treatment strategy to prevent the pathogenesis of IR through ameliorating the incidences of inflammatory responses.

## Background

**Fig. 1:**
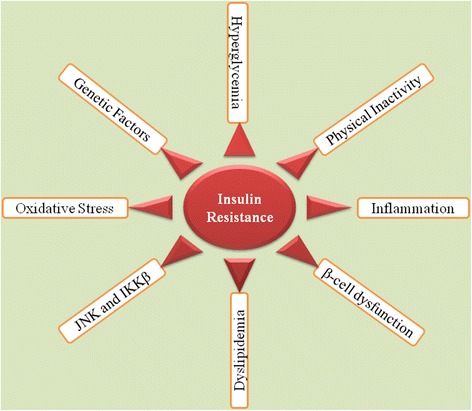
Schematic representation of development of IR. Adopted from Rehman and Akash [5]

Insulin resistance (IR) has long been considered as a major hallmark for the etiology and pathogenesis of type 2 diabetes mellitus (T2DM). Development of IR is mainly associated with low-grade tissue-specific inflammatory responses induced by various pro-inflammatory and/or oxidative stress mediators notably pro-inflammatory cytokines such as interleukin-1 beta (IL-1β), interleukin-6 (IL-6), tumor necrosis factor-alpha (TNF-α), numerious chemokines and adipocytokines [1–3], epigenetic factors, glucolipotoxicity [4], various transcriptional and metabolic pathways (Fig. 1) [5]. Chronic exposure of pro-inflammatory mediators stimulates the activation of cytokine signaling proteins which ultimately block the activation of insulin signaling receptors in β-cells of pancreatic islets [1, 6].

Chronic inflammatory state which is most often characterized with age [7, 8] is indicated by high plasma levels of numerous pro-inflammatory cytokines notably IL-1β, IL-6, CRP, and IL-1β-dependent numerous other cytokines and chemokines [9]. A growing body of evidence has shown that various pro-inflammatory markers such as IL-1β, IL-6, TNF-α, CRP and many chemokines [10–12] are directly or indirectly linked to IR which in turn is more or less commonly accompanied by abnormally elevated levels of pro-inflammatory cytokines, obesity, hypertension and/or glucolipotoxicity [4, 11, 13].

In this article, we have comprehensively summarized the scientific literature and experimental evidences dipicting how inflammatory responses are interlinked with the pathogenesis of IR, including assiciated challeges and last but not least the treatment strategies that may be the opted to counteract development and progression of IR.

## Methods

A comprehensive online English literature was searched via electronic databases including “Med-line”, “PubMed” and “Scopus”. Initially, searched terms like “insulin resistance”, “insulin sensitivity”, “oxidative stress”, “pro-inflammatory mediators and insulin resistance”, “type 2 diabetes mellitus”, “diabetes mellitus”, “cytokines and insulin resistance”, “adipokines and insulin resistance”, “chemokines and insulin resistance”, “endoplasmic reticulum stress and insulin resistance”, “activation of transcriptional pathways and insulin resistance” and “glucolipotoxicity and insulin resistance” used for each term separately. Moreover, we also searched the treatment strategies for insulin resistance. Advanced search was also carried out by combining all search fields in keywords, abstracts and/or titles. Using these search terms, appropriate articles were selected and for a comprehensive review, investigation of literature was further supplemented by searching the referenced articles created by original investigators. Finally, all the selected articles were confirmed for duplications which excluded if it was observed.

## Results and discussion

### Pro-inflammatory mediators and IR

**Fig. 2:**
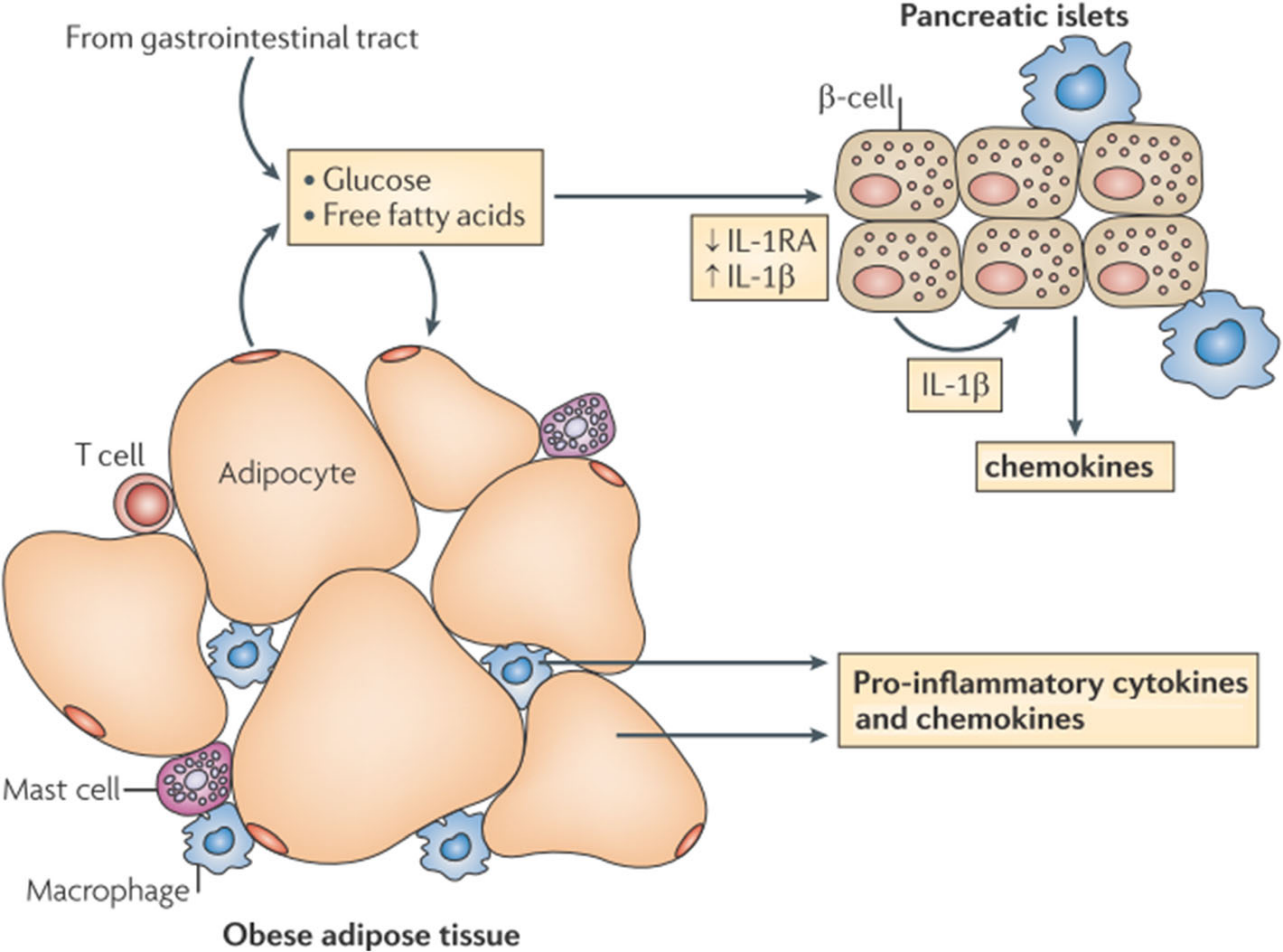
Overnutrition is responsible to elevate the levels of glucose and FFAs in blood which are responsible to induce metabolic stress in β-cells of pancreatic islets and insulin sensitive tissues notably adipocytes (especially in case of obesity). The metabolic stress induced in these tissues activates the release of various pro-inflammatory cytokines notably IL-1β and IL-1β-dependent various other cytokines and chemokines. As a result, immune cells are recruited which contribute the tissue-specific inflammation. Adapted from Donath and Shoelson [12]

Experimental animal models and human epidemiological studies exhibit that IR and inflammation are directly interlinked with each other during the development of T2DM [14, 15]. Pro-inflammatory mediators play crucial role in the development of IR and T2DM through activating various inflammatory responses. Donath and Shoelson [12] have briefly described that how inflammation is developed in T2DM (Fig. 2). In the following sub-sections, we have briefly described the role of various pro-inflammatory mediators in the development of IR.

### IL-β and IR

**Fig. 3:**
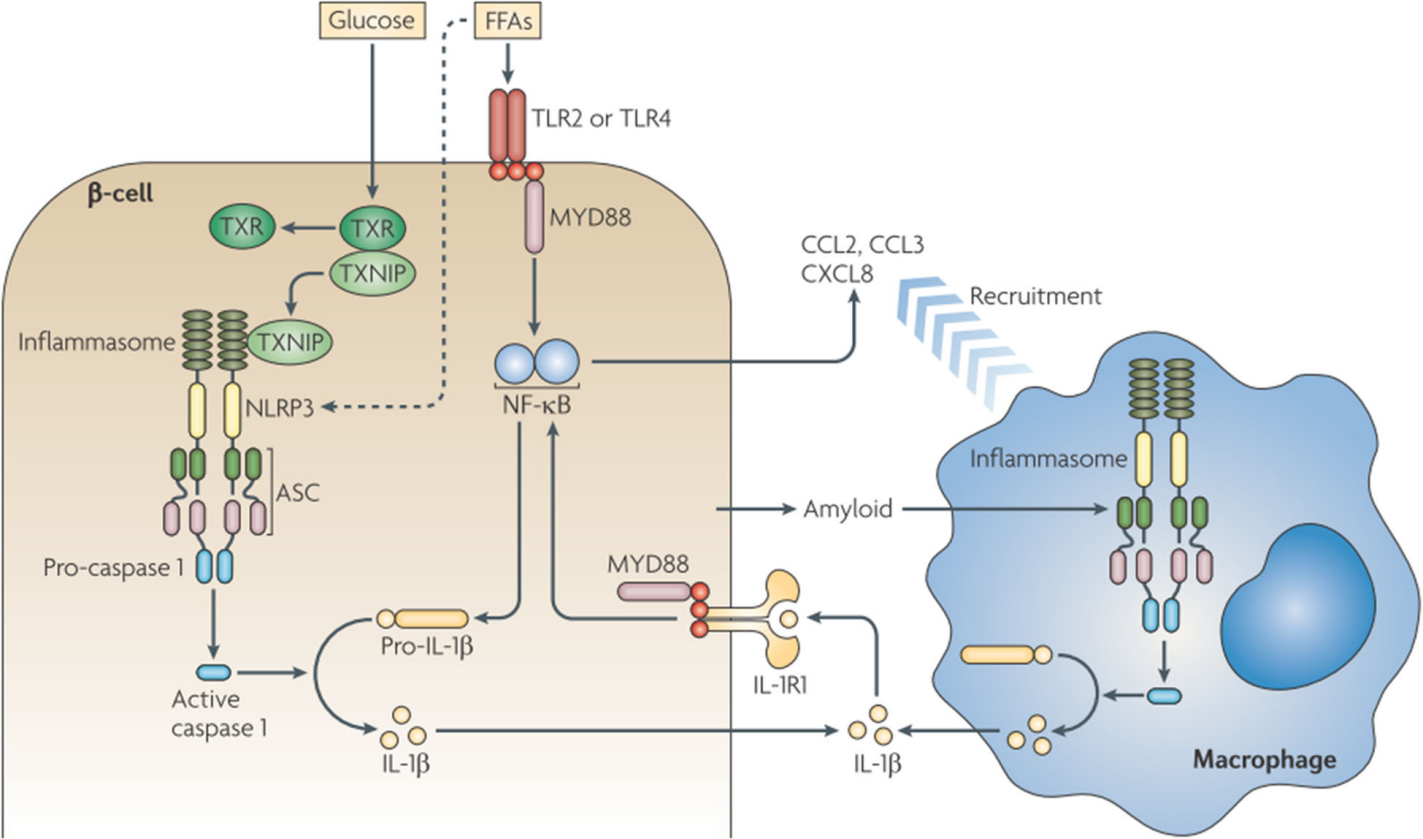
Production of IL-1β-induced inflammation in β-cells of pancreatic islets. Prolonged exposure of FFAs and glucose induce the activation of IL-1β from β-cells of pancreatic islets through the involvement of various transcriptional mediated molecular pathways notably TXNIP, MYD88, NF-κB, TLRs, caspases and inflammasomes. Once IL-1β is activated, it recruits various other pro-inflammatory mediators after binding with its receptor IL-1RI and through the involvement of MYD88 and NF-κB. Adopted from Donath and Shoelson [12]

**Fig. 4:**
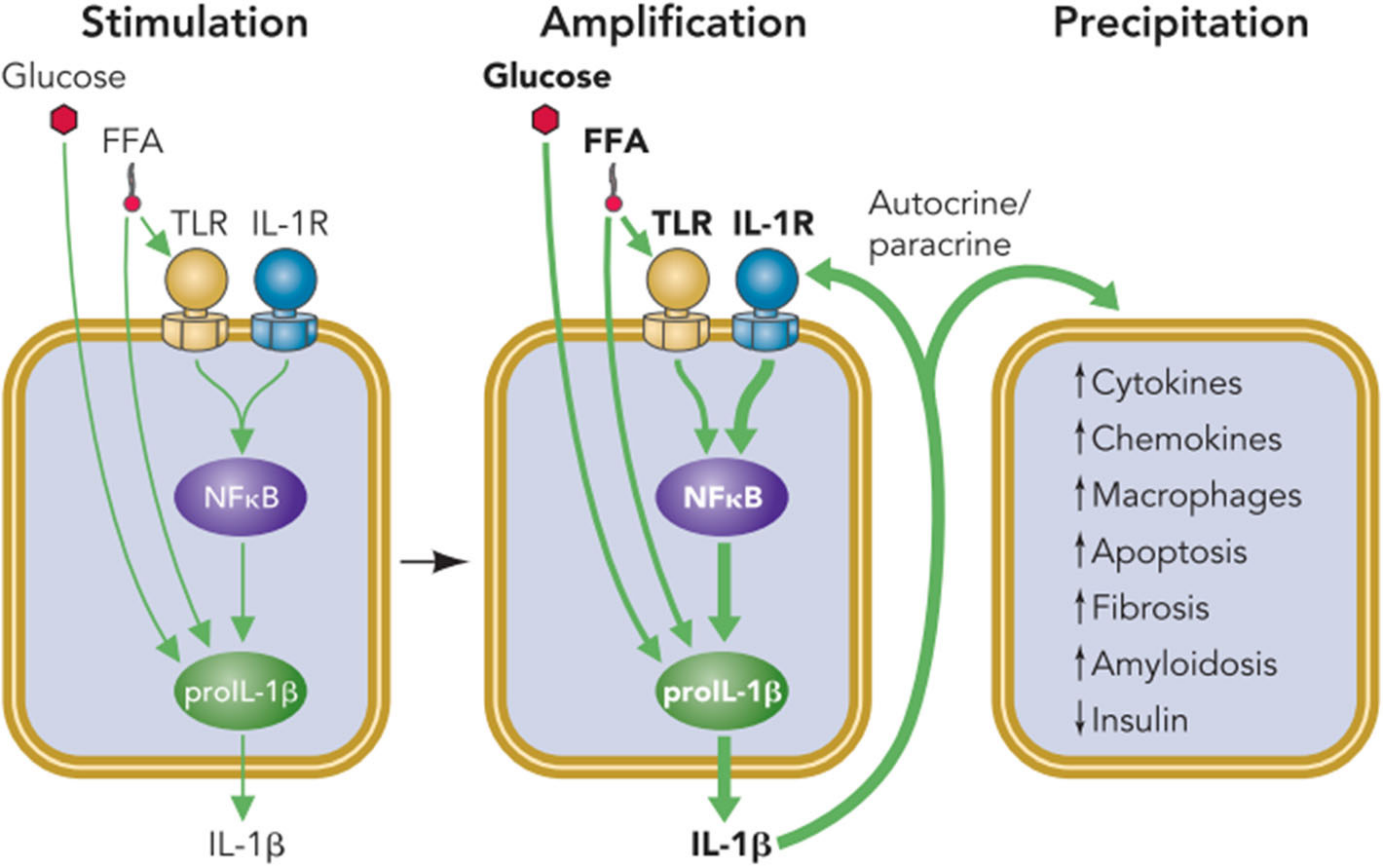
Overnutrition is responsible for elevated levels of glucose and FFAs in blood which entered into the β-cells of pancreatic islets. Initially, these augmented levels of glucose and FFAs induce the expression and release of IL-1β from the β-cells of pancreatic islets (*Stimulation*). Prolonged and/or chronic exposure of glucose and FFAs (also known as metabolic stress) may lead to the activation of IL-1β by activating NF-κB and auto-stimulatory process (*Amplification*). Once, IL-1β is activated and produced, it leads to the recruitment of various other pro-inflammatory cytokines, chemokines, and macrophages (*Precipitation*) which further induces apoptosis, amyloidosis and fibrosis in β-cells of pancreatic islets, and hence impaired insulin secretion occurs whereas, in peripheral tissues, IR is developed due to systemic inflammation. Adopted from Donath et al. [17]

IL-β is a master pro-inflammatory mediator that plays its crucial role to regulate the expression of various other pro-inflammatory cytokines, adipokines and chemokines. It induces inflammation via binding with interleukin-1 receptor type I (IL-1RI) (Fig. 3) and reduces the expression of insulin receptor substrate-1 (IRS-1) at ERK-dependent transcriptional level and ERK-independent post-transcriptional level [16]. Production of IL-1β is mainly regulated by diet-induced metabolic stress (Fig. 4). Experimental studies have been conducted on various experimental animal models to investigate the presence of various inflammatory responses in β-cells of pancreatic islets and peripheral tissues which indicate that IL-β is a master pro-inflammatory mediator that plays its pivotal role to activate numerious other pro-inflammatory cytokines and chemokines [4, 17] through the involvement of various transcriptional mediated pathways. Once, inflammation is produced, it provokes its deleterious effects on β-cells of pancreatic islets due to which impaired insulin secretion occurs in β-cells of pancreatic islets. Likewise, IL-β also plays its decisive role to induce inflammation in peripheral tisuues duw to which the ability of peripheral tissues to utilize insulin in response to glucose is decreased which ultimately leads towards the development of IR in peripheral tissues.

### IL-6 and IR

Aging is associated with increased plasma levels of IL-6 [18] which in turn can be positively correlated with IR [19–22]. The mechanism by which IL-6 induces IR is complicated and versatile [19]. It not only prevents the metabolism of non-oxidative glucose [23, 24], but also suppresses the lipoprotein lipase that consecutively increases the plasma levels of triglycerides [23]. Moreover, IL-6 also activates the suppressor of cytokine signaling (SOCS) proteins [6, 25] which may block the cytokine-mediated transcriptional factor activation of insulin receptor [26]. Signal transducer and activator of transcription 5B (STAT5B) is a protein that belongs to the STAT family of transcription factors. STAT5B is aptly named for its unique ability to act as signal transducer and as transcription factor of insulin receptor [26]. In response to cytokines, STAT5B is phosphorylated by receptor associated kinases [27]. STAT5B activates insulin transcription factor through potentiating the tyrosine kinase by binding with phosphotyrosine 960 of the insulin receptor. The activation of insulin transcription factor is blocked by SOCS proteins which suppresses the activity of tyrosine kinase by significantly competing with STAT5B [19, 27]. SOCS proteins have negative effects on insulin action while IL-6 can activate these SOCS proteins. Therefore IL-6 is considered as an important biomarker for the development of IR [19, 28].

Production of IL-6 is regulated by (IL-1β via activation of interleukin-1 receptor type I (IL-1RI) [29, 30]. Blocking the activity of IL-1RI with suitable anti-inflammatory agent like interleukin-1 receptor antagonist (IL-1Ra) antagonizes the agonistic effects of IL-1β that ultimately leads to the suppression of IL-6 production [4, 31]. Anti-IL-6 receptor antibody and soluble receptor of IL-6 (sIL-6R) have proven to be effective by decreasing the development of IR [32, 33], but this treatment strategy may not be very much effective as production of IL-6 is dependent on the activation of IL-1β and its role in the development of IR cannot be negelected.

### TNF-α and IR

Adipocytes secrete several pro-inflammatory mediators and among them, TNF-α has been proposed to develop a link between IR, obesity and T2DM [34, 35]. Experimental studies conducted on obese animals indicate that the expression of TNF-α is increased in obese animals which modulates the insulin action [36]. TNF-α binds with its receptor and triggers a broad spectrum signaling cascade that results in the activation of various transcriptional pathways such as Nuclear factor kappa-B cells (NF-κB) and Jun NH2-terminal kinase (JNK) [37, 38]. Once, NF-κB and JNK are activated, they phosphorylate serine 307 in IRS-1 which result in the impairment of IR-mediated tyrosine phosphorylation of IRS-1 [37]. Recently, it has been found that serum level of TNF-α is positively correlated with the pathophysiology of IR [35, 39] which exhibit that TNF-α is also a main causative factor that contributes the development of IR.

### Adipokines and IR

**Fig. 5:**
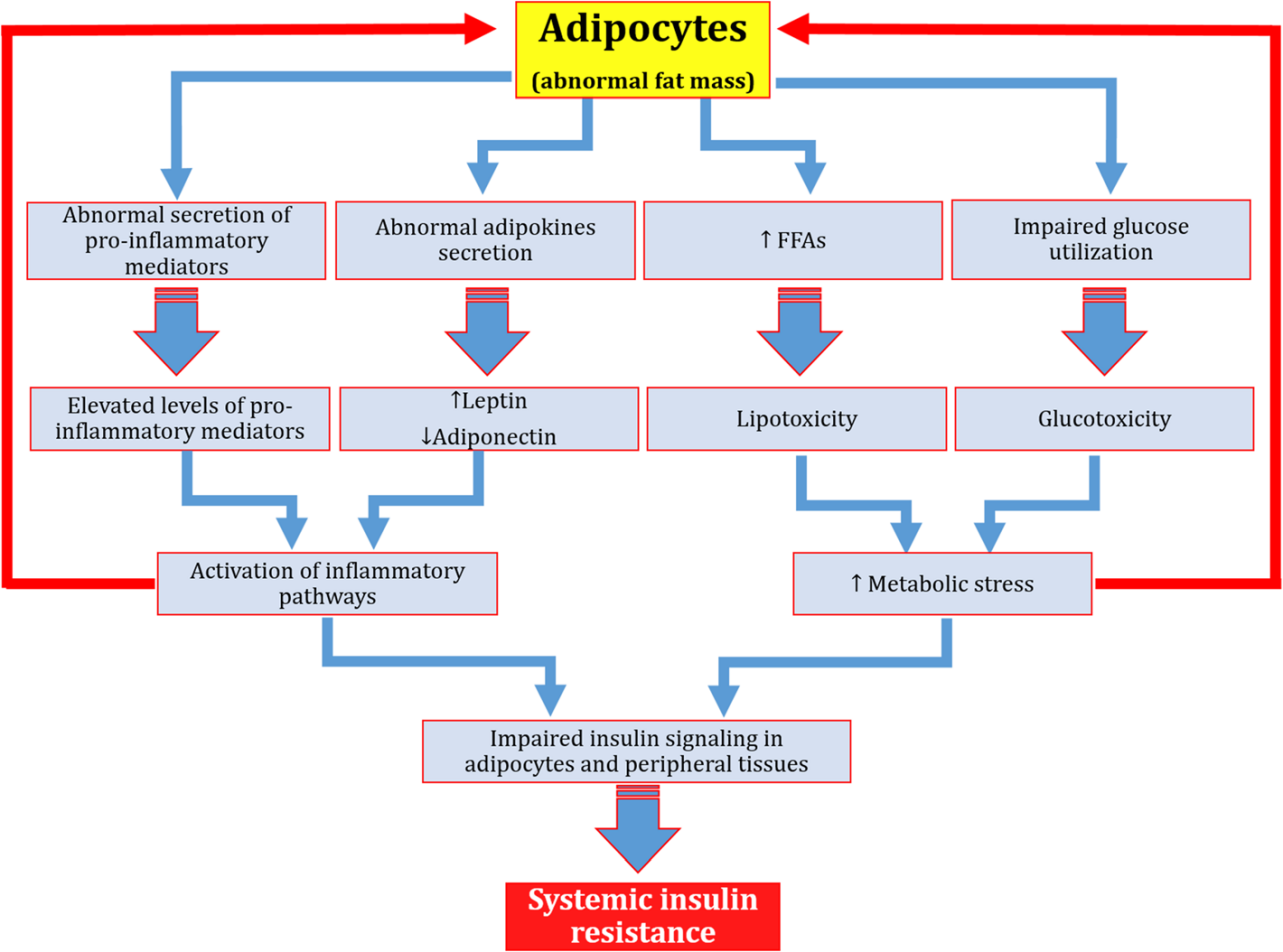
Schematic representation of adipocytokines-induced IR. Glucolipotoxicity and induction of inflammation in adipocytes are responsible to make the adipocytes abnormal. Once adipocytes are injured, glucose utilization is decreased in adipocytes and levels of FFAs are abnormally increased due to which metabolic stress in adipocytes is increased which ultimately leads to the abnormal secretion of various pro-inflammatory mediators and adipocytokines. Abnormal secretion of these pro-inflammatory mediators and adipocytokines activate various inflammatory pathways which impairs the phosphorylation of various insulin signaling pathways in adipocytes and/or peripheral tissues due to which systemic IR is developed

Previously, it has been thought that adipose tissues are the main site for energy storage and/or supply, but now, it has been recognized that adipose tissues are actively involved in communitation with other tissues due to which it is considered as an active endocrine organ [40]. Therefore, it has been deliberated that adipose tissues are the major endocrine organ which have the ability to produce variety of adipose-derived mediators that are activitely involved to regulate the energy metabolism and insulin sensitivity [41]. The most important adipose-derived mediators are FFAs and adipokines. Adipokines include large number of pro-inflammatory mediators which include leptin, TNF-α, IL-6, tissue inhibitor of metalloproteinases (TIMP-1) adiponectin, retinol-binding protein (RBP-4) and monocyte chemotactic protein (MCP-1) [42, 43]. It has been evidenced from several experimental studies that there is a strong correlation between the mass of adipose tissues and development of IR (Fig. 5) in peripheral tissues of diabetic patients [44, 45]. Adipose tissue’s mass in obesity and lipodystrophy becomes abnormal which results in the development of IR in peripheral tissues. Adipokines indicate the chronic low-grade inflammation in adipose tissues [46] and have been considered as emerging biomarkers for insulin sensitivity and/or resistance. IL-6, TNF-α, MCP-1, TIMP-1, RBP-4, and leptin are considered as pro-inflammatory cytokines which are responsible not only for the induction for local inflammation in adipocytes, but may also induce systemic inflammation after entering into the blood stream [4, 47, 48]. Adiponectin is the only adipokine that acts as anti-inflammatory cytokine and has the ability to ameliorate the deleterious effects of IL-6, TNF-α, MCP-1, TIMP-1, RBP-4, and leptin which are known to be produced in adipose tissues [11]. It has also been found that the level of adiponectin is downregulated in obesity and is positively associated with insulin sensitivity [49, 50]. The imbalance between leptin and adiponectin may result in the development of systemic IR.

### Chemokines and IR

**Fig. 6:**
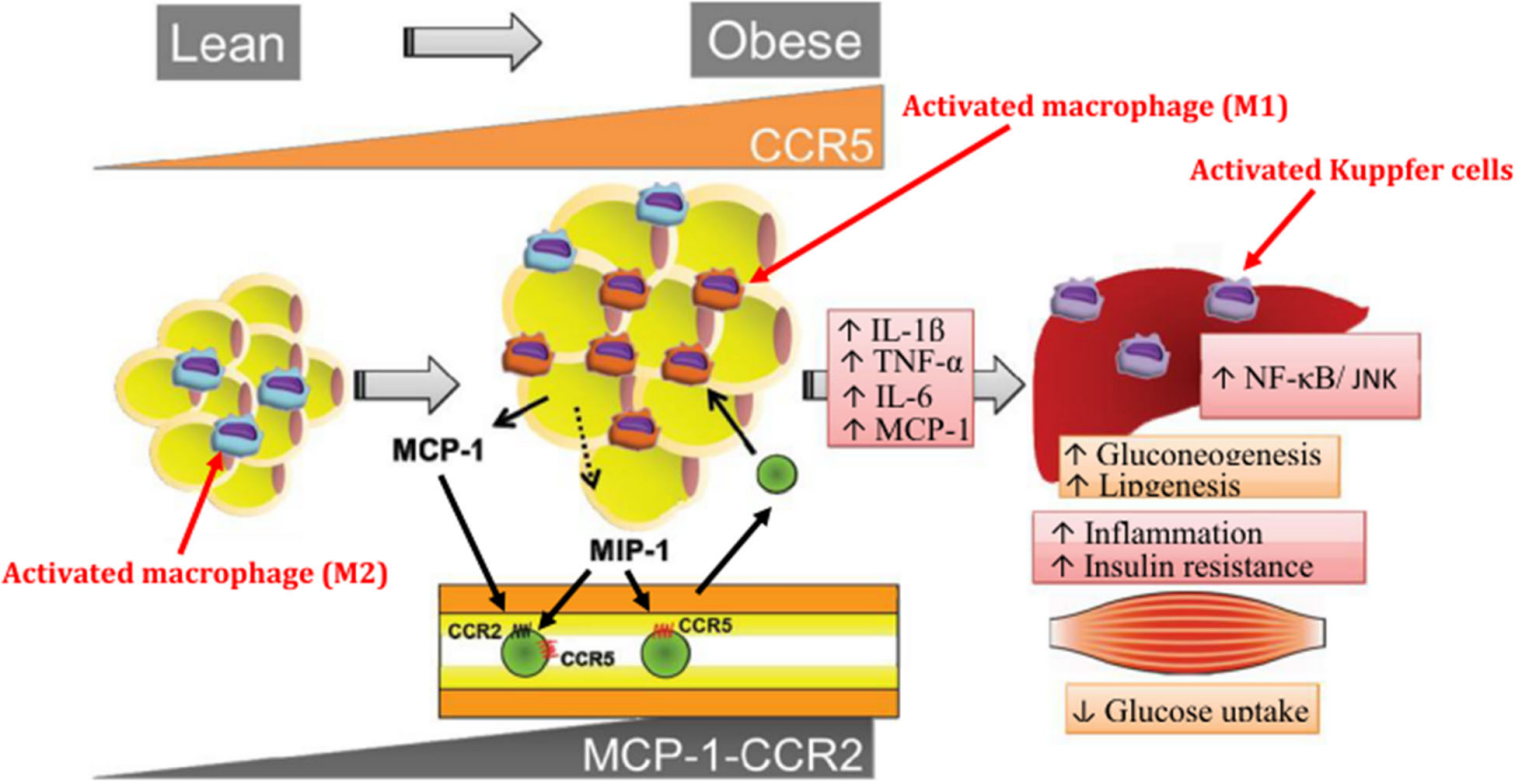
Chemokines-induced IR. M2 macrophages in lean state, maintain the insulin sensitivity in adipose tissues whereas, due to overnutrition, adipose tissues initiates the secretion of MCP-1 which leads to the recruitment of circulating monocytes in adipocytes. CCR2 macrophages are accumulated in obese adipocytes and presumably maintain the inflammation by recruiting M1 macrophages in obese adipocytes. While on the other side, CCR5-adipose tissue macrophages (ATM) also infiltrate from the obese adipocytes and promote the inflammatory responses by involving ATM recruitment and producing various pro-inflammatory mediators notably TNF-α, IL-6, and IL-1β in conjunction with other infiltrated immune cells and adipokines. After production, these pro-inflammatory mediators induce IR in adipocytes and peripheral tissues through activation of several transcriptional pathways such as JNK and NF-κB. Adapted from Xu et al. 2015

**Fig. 7:**
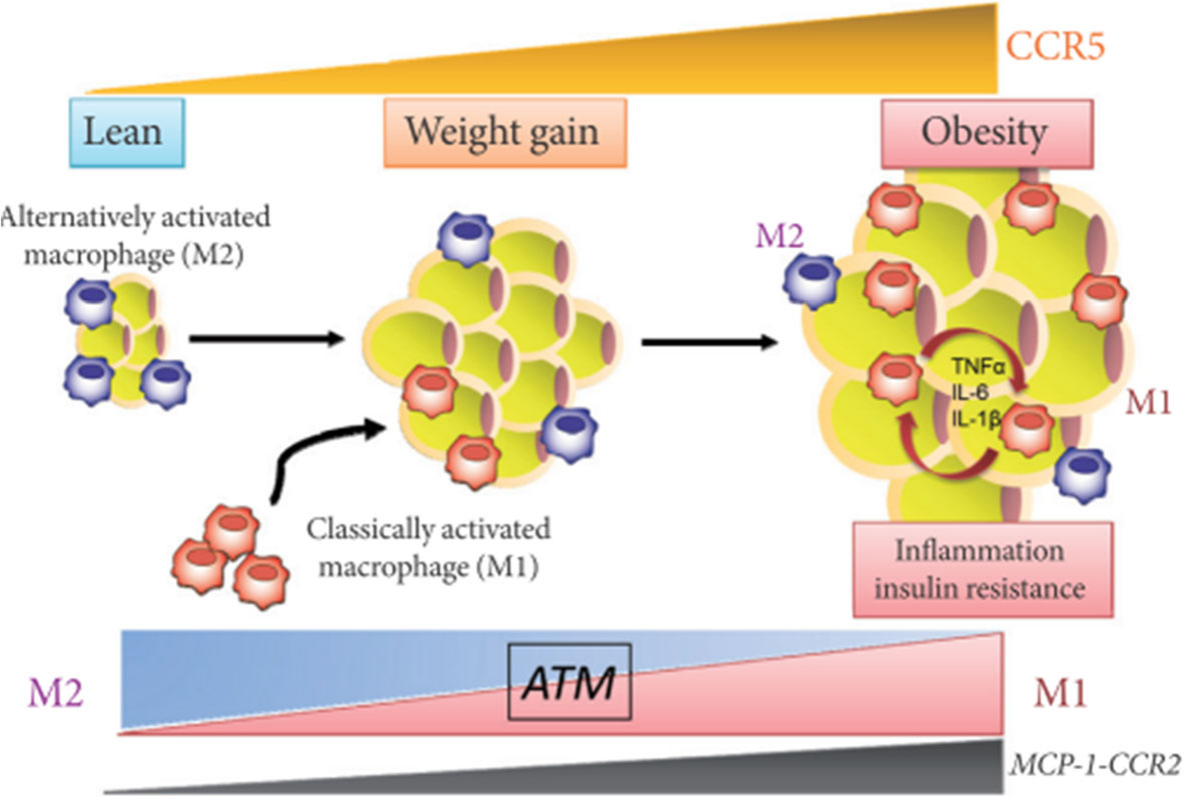
C-C motif chemokine receptor 5 (CCR5) promotes obesity-induced inflammation and IR. Recently, it has been found that the expression of CCR5 and its ligand MCP-1 is significantly increased in white adipose tissues (WAT) and its accumulation is increased in adipose tissue macrophages (ATM) in WAT of obese mice and provides a novel link between inflammation and IR in adipocytes by stimulating the production of various pro-inflammatory cytokines and chemokines. Adopted from Ota [51]

Chemokines are an important class of pro-inflammatory mediators. Their production is dependent on the activation IL-1β and various transcriptional pathways [4]. Up till now, various chemokines have been discovered, among which the most important are MCP-1, MCP-2, MCP-3, MCP-4, CCL2, MIP-1α and MIP-1β [51]. Several studies have reported that MCP-1 and CCL2 deficient mice prevented high fat diet-induced IR [52, 53]. Moreover, overexpression of MCP-1 in adipose tissues was also observed to be responsible for the increase in adipose tissue macrophages and induction IR [52, 54]. Obesity is the state of chronic low-grade inflammation which is linked to the development of local and/or systemic IR. It has been found that chemokines play crucial role for the development of IR and T2DM (Fig. 6) [55]. Among various receptors for chemokines, CCR2 and CCR5 are the most important receptors that play decisive role in the pathogenesis of IR [56] in adipose tissues (Fig. 7). It has been found that adipocytes secrete CCR2 in an inactive form. After activation, CCR2 induces the expression of various inflammatory genes and impaires the uptake of insulin-dependent glucose uptake. Moreover, adipocytes can also secrete CCL2 and CCL3 which act as a potent signal for the recruitment of macrophages. The upregulation of CCL2 and CCL3 from adipocytes may contribute to the development of IR in adipose and peripheral tissues [57]. The above mentioned studies highlight the crucial role of various chemokines in the development of IR along with other pro-inflammatory mediators.

### CRP and IR

CRP has been considred as one of the most important human acute phase protein that correlates with development of IR [58, 59]. CRP is a systemic inflammatory biomarker and has been considered as one of the major causative factor for the development of T2DM [60]. It has been evidenced that elevated levels of CRP not only reflect the induction of local inflammation, but also predict the pathogenesis of tissue-specific IR [61]. Several studies have found that strong relationship exists between levels of CRP and development of IR [61–63] which indicates that besides other pro-inflamamtory mediators, CRP also actively plays its pivotal role for the pathogenesis of IR by inducing local and/or systemic inflammation.

### Oxidative stress and IR

**Fig. 8:**
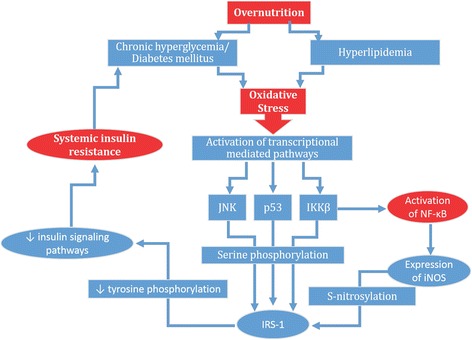
Mechanism of oxidative stress-induced IR: Chronic exposure of hyperglycemia and hyperlipidemia due to over nutrition leads to the production of oxidative stress via activation of reactive oxygen species. Once, oxidative stress is produced within the body, it leads to the activation of various transcriptional mediated pathways such as p38, JNK, IKKβ and/or NF-κB. IKKβ also induces the activation of NF-κB. p38, JNK and IKKβ, further activates the serine phosphorylation of insulin receptor substrate-1 (IRS-1). While on the other side, NF-κB also activates the expression of iNOS which also induces the S-nitrosylation of IRS-1. Both S-nitrosylation and serine phosphorylation of IRS-1 suppress the tyrosine phosphorylation of insulin signaling pathways which ultimately results into the induction of IR in liver, adipocytes and skeletal muscles

**Fig. 9:**
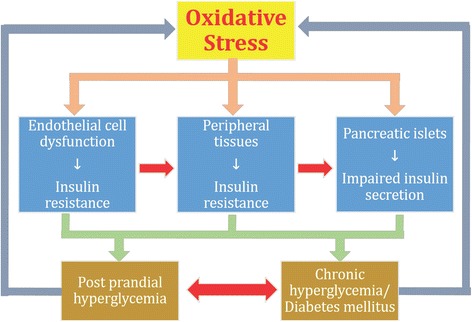
Impact of oxidative stress on vital organs of the body. β-cells of pancreatic islets, adipocytes and peripheral tissues are more susceptible to the damaging effects of oxidative stress. Oxidative stress independently exhibit its hazardous effects on these organs due to which impaired insulin secretion occurs in β-cells of pancreatic islets and IR develops in adipocytes and peripheral tissues. Impaired insulin secretion and IR lead to the development of post prandial hyperglycemia and overt T2DM both of which also acts as feedback mechanism for the development of oxidative stress

Overnutrition increases the cellular overload of glucose and FFAs which in turn increases the oxidative stress (Fig. 8). Peripheral and adipose tissues protect themselves from the damaging effects of oxidative stress producing resistance to the action of insulin by preventing the penetration of glucose and FFAs into the cells. Oxidative stress is because of imbalance between the production of reactive oxygen species (ROS) and anti-oxidative defense mechanism against the production of ROS. β-cells of pancreatic islets, adipocytes and peripheral tissues are more vulnerable to the damaging effects of oxidative stress (Fig. 9). Several mechanisms are involved to influence the balance between ROS and anti-oxidant defense mechanisms including activation of stress-signaling pathways such as JNK pathway [64] and transcriptional mediated pathways such as NF-κB [65]. JNK and NF-κB pathways decrease the insulin-mediated glucose uptake by tissues and insuling signaling [66–68], that ultimately induces IR (Fig. 8). Moreover, the activations of JNK and NF-κB pathways is also associated with the upregulation of various pro-inflammatory mediators such as TNF-α, IL-6, and CRP. It has also been reported that oxidative stress-indcued activation of NF-κB pathway may also be associated with endothelial dysfunction that can lead to the induction of IR [69, 70], but anti-oxidant therapy may act as a potential strategy to prevent the induction of IR-associated with endothelial dysfunction [71]. The growing body of evidence indicate that oxidative stress is a common pathogenic factor that leads to the development of tissues-specific IR. The results of experimental studies indicate that what happens in peripheral tissues also occur in the β-cells of pancreatic islets and endothelial cells to compensate the systemic oxidative stress.

### Endoplasmic reticulum stress and IR

Endoplasmic reticulum stress (ERS) is another mechanism that palys crucial role for the development of IR in adipocytes and peripheral tissues. ERS just like oxidative stress, is produced by the activation of JNK and inhibitory phosphorylation of IRS-1 in adipose tissues and liver [72] and induces the pathogenesis of IR in endothelial cells. It has been found that ER is a major site for the production of various proteins such as insulin biosynthesis and act as a place for the lipid and sterol synthesis [73]. Any kind of abnormality that occurs in ER may lead to the development of ERS which also contribute to induce tissue-specific IR. It has been revealved from experimental studies that some anti-diabetic agents alos modulate the ERS during the treatment of T2DM [74] which offer a new therapeutic target for the treatment of ERS-inducced IR and T2DM.

### Activation of transcriptional pathways and IR

NF-κB is a sequence-specific transcriptional mediated factor that primarily regulates various inflammatory responses [75] and IκB kinase β (IKK-β) is a central coordinator for these inflammatory responses through the activation of NF-κB [76]. IKK-β activates NF-κB through phosphorylation of IKK-β [77, 78] and thereafter, NF-κB mediates the stimulation of numerous pro-inflammatory mediators such as IL-1β, IL-6, and TNF-α [76, 78]. Once these pro-inflammatory cytokines are activated, they ultimately lead to cause IR [2, 14, 79, 80]. Therefore, NF-κB and IKK-β are considered to be involved in the pathogenesis of IR [81, 82]. IKK-β induces inflammatory responses in hepatocytes which massively increase the production of pro-inflammatory cytokines [83]. These pro-inflammatory cytokines then enter into the blood stream to cause IR in other tissues [81].

Various studies have investigated that nonsteroidal anti-inflammatory drugs (NSAIDs) such as cyclooxygenase inhibitors (aspirin and salicylates) can significantly inhibit the activation of NF-κB and IKK-β [84] in rodent models and humans [84, 85]. These studies suggest that NSAIDs may exhibit their anti-inflammatory effects on myeloid cells rather than in muscle or fat. Expression of IKK-β in myeloid cells significantly suppresses the activation of pro-inflammatory cytokines that promote IR [81]. In the following sub-sections, role of various transcriptional pathways in the pathogenesis of IR has been briefly described.

### Activation of Toll like receptors and IR

**Fig. 10:**
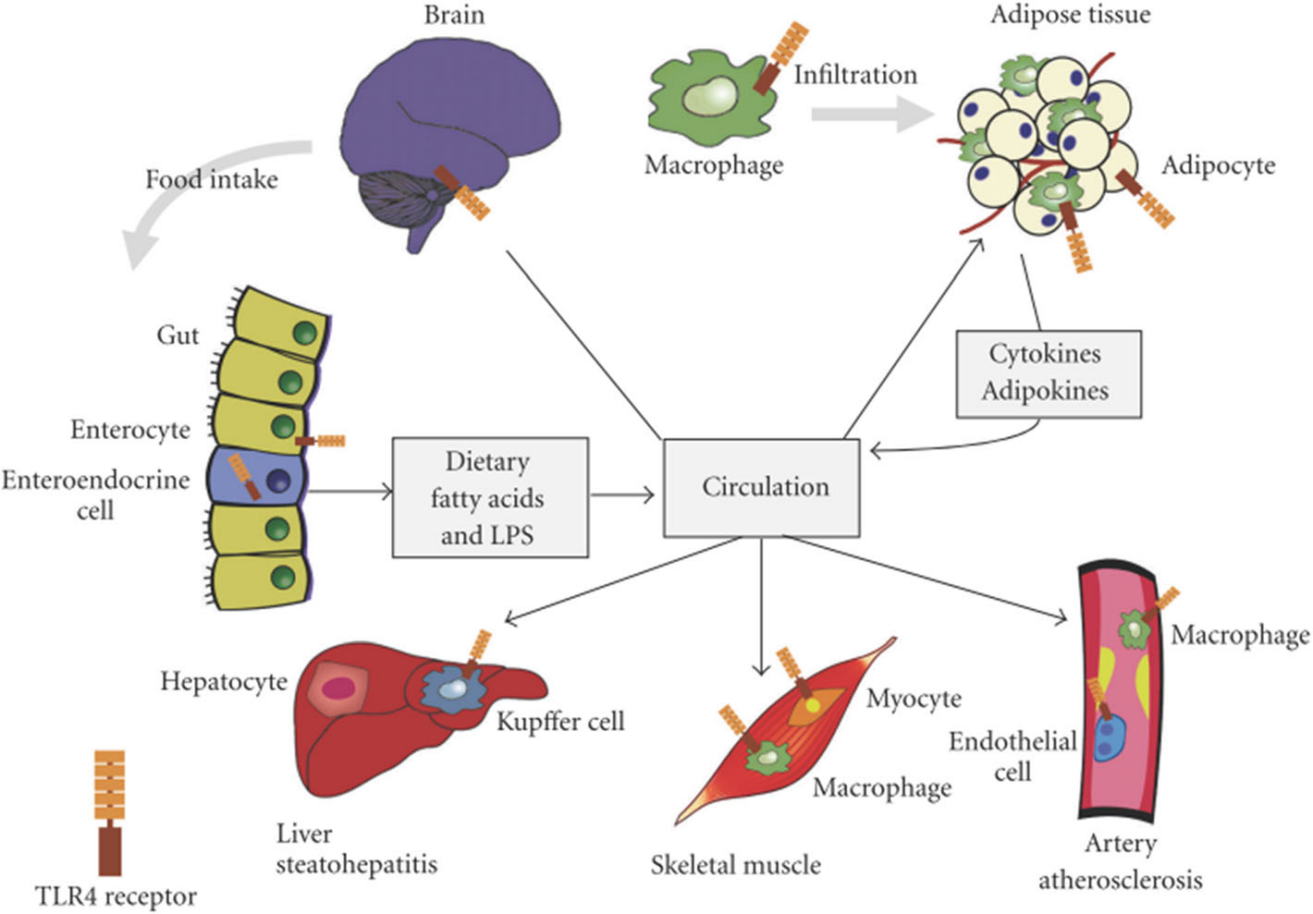
Expression TLR4 in integrated tissues and organ systems of the body that regulate the insulin sensitivity. Toll-like receptor 4 (TLR4) present in adipocytes, initiates the inflammatory responses that release various pro-inflammatory mediators. Once, produced, these mediators are entred into the blood stream and thereby promote IR. Liver-resident macrophages known as Kupffer cells are made up of 10% of the cells in liver and 80%–90% of all tissue macrophages within the body. TLR4, expressed on Kupffer cells and other liver cell components, regulates the various inflammatory responses in liver. TLR4, expressed in skeletal muscles, has been shown to regulate the substrate metabolism in muscle, favoring glucose oxidation in the absence of insulin. Hypothalamus and mesolimbic area are important sites that modulate the energy expenditure, pancreatic β-cell function and IR in peripheral tissue. Expression of TLR4 in hypothalamus potentiates various inflammatory responses that contribute to the pathogenesis of IR. Adopted from Kim and Sears 2010

**Fig. 11:**
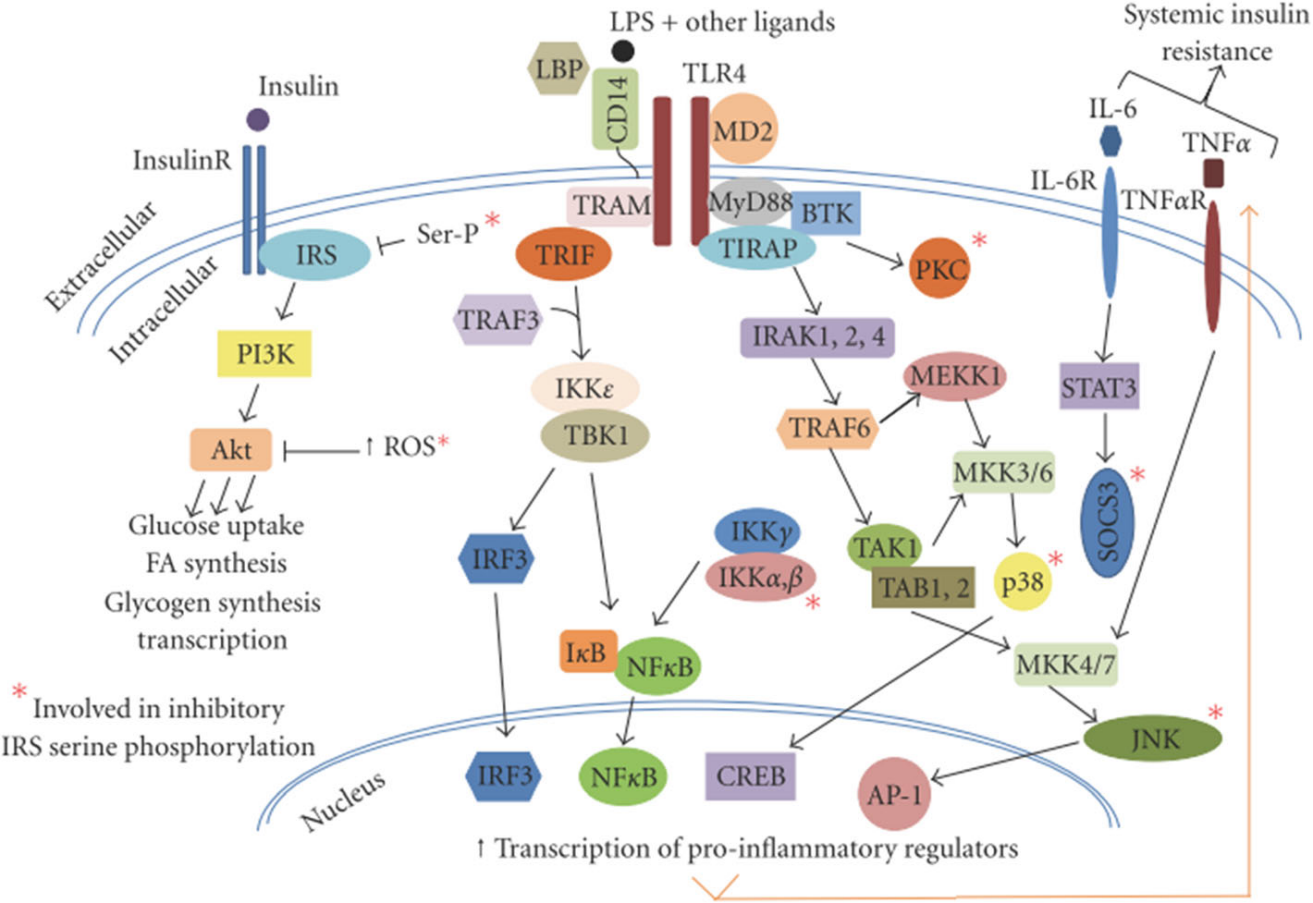
Schematic representation of TLR4 signaling cascades. Signal transduction of TLR4 through the activation of MyD88/TIRAP and TRAM/TRIF pathways, leads to potentiate the innate immune responses and inhibit signal transduction of insulin, primarily through serine phosphorylation of IRS. Adopted from Kim and Sears 2010

IR leads to the increased production of insulin from β-cells of pancreatic islets and as result, compensatory hyperinsulinemia within the body occurs. Toll like receptors (TLRs) are the important modulators of IR and its comorbidities. Chronic inflammation plays a crucial role in variety of insulin resistant states [86, 87] in which various signaling pathways are activated that directly interfere with the normal functioning of the key components of insulin signaling pathways [88]. Among various pathways, activation of TLRs imparts crucial role for the generation of inflammation. There are two main types of TLRs i.e. TLR2 and TLR4. TLR4 is an extracellular cell surface receptor that is expressed in β-cells of pancreatic islets, brain, liver skeletal muscle and adipose tissues (Fig. 10) [89]. In nomal conditions, TLR4 regulates insulin sensitivity in these tissues, but the activation of TLR4 directly dampen the insulin action through the activation of various pro-inflammatory mediators and ROS, indirectly generates the activation of various pro-inflammatory mediators by inducing various signaling cascades and transcriptional factors notably MyD88, TIRAP, TRIF, IKKs and JNKs that causes the activation of innate immune responses which ultimately leads to the development of IR (Fig. 11) [89]. TLR4 plays this role primarly in coordination with the phosphorylation of IRS serine.

Lipopolysaccharide (LPS) and its endotoxic moiety have been reported to be the potential activators of TLR4 (Fig. 11). LPS is composed of oligosaccharides and acylated saturated fatty acids (SFAs). Besides LPS, SFAs have also been reported to be the activator of TLR4. The expression and signaling of TLR4 are regulated mainly by the adiponectins. Several studies have reported that adiponectin can inhibit LPS-induced activation of TLR4 through the involvement of AMPK, IL-10, and heme oxygenase-1 [90–92]. Other regulators of TLR4 are peroxisome proliferators-activated receptor gamma (PPARγ) and sex hormones [93, 94]. Taking together, TLR4 is a molecular link for pro-inflamatory mediators, different body organs, and several transcriptional pathways and cascades that modulate the innate immune system by regulating the insulin sensitivity. In the proceeding sub-sections, role of TLR4 expression in various vital organs of the body for the pathogenesis of IR has been described.

### TLR4 expression in adipose tissues

Despite of having the ability to act as storage depot for excess calories, adipose tissues secrete large number of hormones, pro-inflammatory cytokines and chemokines that directly influence the metabolism (Fig. 10). Adipose tissues consist of adipocytes, preadipocytes, macrophages, lymphocytes and endothelial cells. Only adipocytes and macrophages are known to release various pro-inflammatory cytokines (IL-1β, IL-6, and TNF-α) and chemokines (such as MCP-1) that potentiate inflammation in several tissues after being released into the systemic circulation [95]. Besides this, adipocytes are also a rich source of two important hormones namely leptin [96, 97] and adiponectin [98]. Adiponectin, having anti-inflammatory properties, promotes insulin sensitivity whereas, leptin having inflammatory properties, impairs insulin sensitivity in adipocytes [87]. Several factors such as oxidative stress, increased FFAs flux and hypoxia that are associated with inflammation can induce IR in adipose tissues [87]. TLRs present in adipose tissues are directly activated by the nutrients [99, 100] which play a key role for the initiation of inflammatory responses which ultimately promotes IR in these tissues [100–104]. In experimental studies, it has been found that LPS-resistant strains of mice with loss-of function (C3H/HeJ mice) and deletion (C57BL/10ScN mice) mutations in TLR4 gene [105] resulted in imporved insulin sensitivity with increased rate of glucose utilization in skelectal muscle and adipose tissues [100, 101]. Nutritional fatty acids can activate the expression of TLR4 in adipocytes that play crucial role for the activation of various pro-inflammatory mediators and transcriptional mediated pathways which ultimately lead to the development of IR in adipocytes.

### TLR4 expression in skeletal muscle

Skeletal muscles have marked significance to regulate the normal glucose homeostasis and development of IR as these are the primary site for insulin-induced glucose uptake and utilization in peripheral tissues. 75% of the insulin-induced glucse utilization occurs in skeletal muscles under normal physicological conditions [106] which is markedly reduced in hyperinsulinemic and obese patients.

Skeletal muscles contain myocytes and macrophages in which TLR4 receptors are expressed (Fig. 10). Signal transduction of TLR receptors is an underlying mechanism for the development of IR and chronic inflammation in skeletal muscles [107]. TLR4 expression in skeletal muscle is associated with severity of IR and skeletal muscle metabolism. The mechanis in the development of IR in skeletal muscles may include the direct effects of intramyocellular FFAs metabolites in skeletal muscles, macrophages and paracrine effects of adipocytes. Recently, it has been experimentally confirmed that disruption of TLR4 expression prevents SFA-induced IR in TLR mutant mice and improves IRS-1 tyrosine phosphorylation and insulin-stimulated glucose uptake. Moreover, disruption of TLR4 expression has also shown to decrease the JNK1 phosphorylation and IRS-1 serine phosphorylation [100, 104].

### TLR4 expression in liver

Liver is the major and vital organ of the body which is composed of heterogenous types of cells notably hepatocytes, immune cells, kupffer cells and endothelial cells. Kupffer cells are known as liver-resident macrophages which compose of 10% cells in the liver and 90% of all tissue macrophages in body. Due to their localization at sinusoids, kupffer cells are in close contact with circulating cytokines, lipids, hormones and postprandial LPS, and hence, kupffer cells are important mediators of inflammation within the liver. TLR4 expressed on kupffer cells in the liver (Fig. 10), are responsible to modulate the activity of pro-inflammatory mediators which are induced by IR, fructose- and high-fat diet-induced hepatic steatosis [100, 102, 108, 109]. It has been found that activated levels of pro-inflammatory AP-1 and NF-κB in liver are directly correlated with IR and oxidative stress [110]. TLR4 signaling pathway is strongly associated with IR as, it has been found that acute treatment of LPS inhibits the production of hepatic glucose via activation of TLR4 signaling pathway and induces IR in liver [111].

### TLR4 expression in β-cells of pancreatic islets

Several TLRs such as TLR2, TLR3 and TLR4, are also expressed in β-cells of pancreatic islets [112]. Signal transduction of TLRs in β-cells of pancreatic islets is mainly associated with inflammation in β-cells of pancreatic islets [113–116]. Distruction and malfunctioning of β-cells of pancreatic islets may lead to insufficient secretion of insulin in both types of DM. TLR4 expression in β-cells of pancreatic islets, is induced by the toxic levels of glucose and FFAs in the blood, cytokine signaling, and/or ER stress within β-cells of pancreatic islets [117]. Expression of TLR4 in pancreatic islets may lead to impaired insulin secretion and promote β-cell apoptosis [118].

### TLR4 expression in brain

Brain itself palys a central role to regulate glucose homeostasis and metabolism. In brain, hypothalamus and mesolimbic sites have been considered as important areas that are actively involved in the regulation of insulin sensitivity in peripheral tissues and β-cells secretory functions of pancreatic islets [119]. TLR4 expression is widely distributed in the body (Fig. 10), but signal transduction of TLR4 in CNS affects the intake of food and contribute to the development of obesity [100] and activates various pro-inflammatory signalling pathways such as activation of JNK1, MyD88 and NF-κB pathways in hypothalamus that ultimately contribute to the development of tissue-specific IR [120–122].

### TLR4 expression in endothelial cells

Vascular endothelial dysfunction is a major complication for induction of IR and pathogenesis of T2DM. At molecular level, excess amount of nutrient is interlinked with IR through the activation of transcriptional mediated pathways such as IKKβ and NF-κB [83, 123]. Augmented levels of FFAs are associated with generation of inflammation and induction of IR in endothelial cells [124, 125]. IKKβ and NF-κB are transcriptional mediators of inflammation and TLR4 is implicated as a mediator of IKKβ and NF-κB [100, 126]. TLR4 receptors are also expressed in endothelial cells and expression of TLR4 via LPS-stimulated IKKβ and NF-κB activation contributes the dysfunctioning of endothelial cells [126]. Activation of TLR4 via FFAs can trigger the cellular inflammatory responses in endothelial cells [127, 128] whereas, whole body deletion of TLR4 expression has shown to prevent high-fat diet-induced vascular inflammation and IR in mice [129, 130]. Similarly, activation of TLR4-dependent IKK and NF-κB indicated impaired insulin signaling and NO production in endothelial cells [131]. The growing evidence implicates that TLR4 is the major causative factor to induce IR in endothelial cells via activation of various transcriptional mediated pathways and inflammation in endothelial cells.

### AMPK and IR

**Fig. 12:**
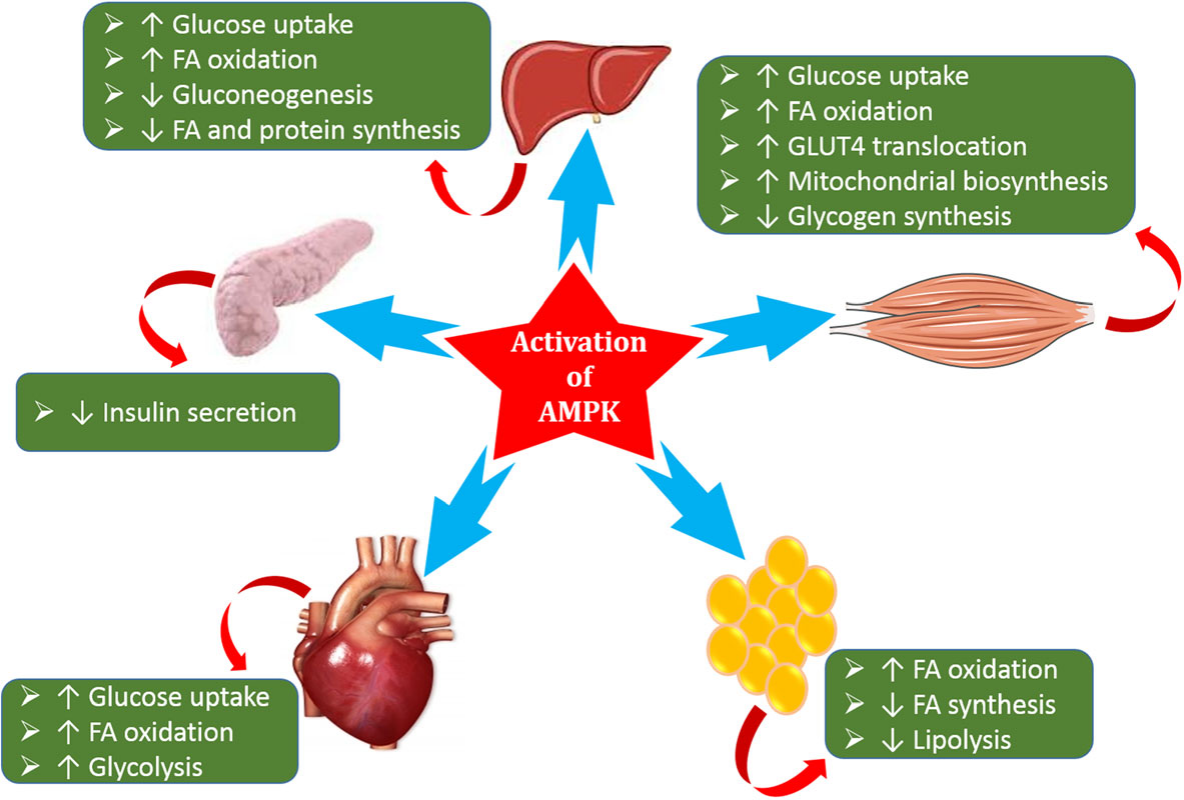
Schematic representation of effect of AMPK activation on various body organs

AMP-activated protein kinase (AMPK) is an enzyme that is most commonly known as master regulator of energy metabolism [132] and its activation is based on the energy level of the body. Upon activation, AMPK resotres the energy levels of the body by stimulating various processes in different body organs (Fig. 12) that are responsible to generate the energy [133–136]. AMPK plays a crucial role between adipose and peripheral tissues, and interferes various metabolic and secretory functions [137] that are responsible for normoglycemia and glucose homeostasis (Fig. 12). In adipocytes, adipokines exhibit their metabolic effects by activating AMPK which result in the increased β-oxidation in peripheral tissues. There is strong correlation between development of IR and generation of inflammation induced by oxidative and/or ER stress, and glucolipotoxicity. It has been evidenced that activation of AMPK is suppressed by the generation of inflammation [138–140] and/or glucolipotoxicity which leads to the development of IR [141]. Activation of AMPK in peripheral tissues enables skeletal muscles to cope with elevated levels of FFAs. Keeping in view the active role of AMPK in energy metabolim, it has been found that AMPK activation improves insulin sensitivity and glucose homeostasis. IR is a major hallmark for the pathogenesis of T2DM however, AMPK activation can prevent the pathogenesis of IR and development of T2DM.

### Activation of protein kinases and IR

Protein kinase C (PKC) and inhibitor kB kinase (IKK) are the two main important kinases that play crucial role in pro-inflammatory mediators-induced inflammatory processes in adipocytes and peripheral tissues underlying the development of systemic IR [142–144]. IKK induces IR in peripheral tissues by suppressing the insulin signaling and activating NF-κB [125, 145]. Inhibition of IKK activation prevents the secretion of adipokines from adipocytes and improves insulin sensitivity in adipocytes and peripheral tissues [81, 146, 147].

### NF-κB and IR

NF-κB is a transcriptional mediated pathway that plays its crucial role in the transcription of signals for te production and release of various pro-inflammatory mediators. Most importantly, NF-κB plays active role to regulate IL-1β (Fig. 12). Metabolic and/or oxidative stress induces various kinases such as IKKβ and JNK [81, 83, 123] which play a key role to activate NF-κB and impairs insulin signaling pathways that ultimately leads to the development of IR (Fig. 8). Once activated, NF-κB targets serval genes to potentiate the release of various pro-inflammatory mediators in adipose tissues and liver [81, 83, 123]. These pro-inflammatory mediators that are produced in response to NF-κB activation induce tissue-specific IR.

### Glucolipotoxicity and IR

**Fig. 13:**
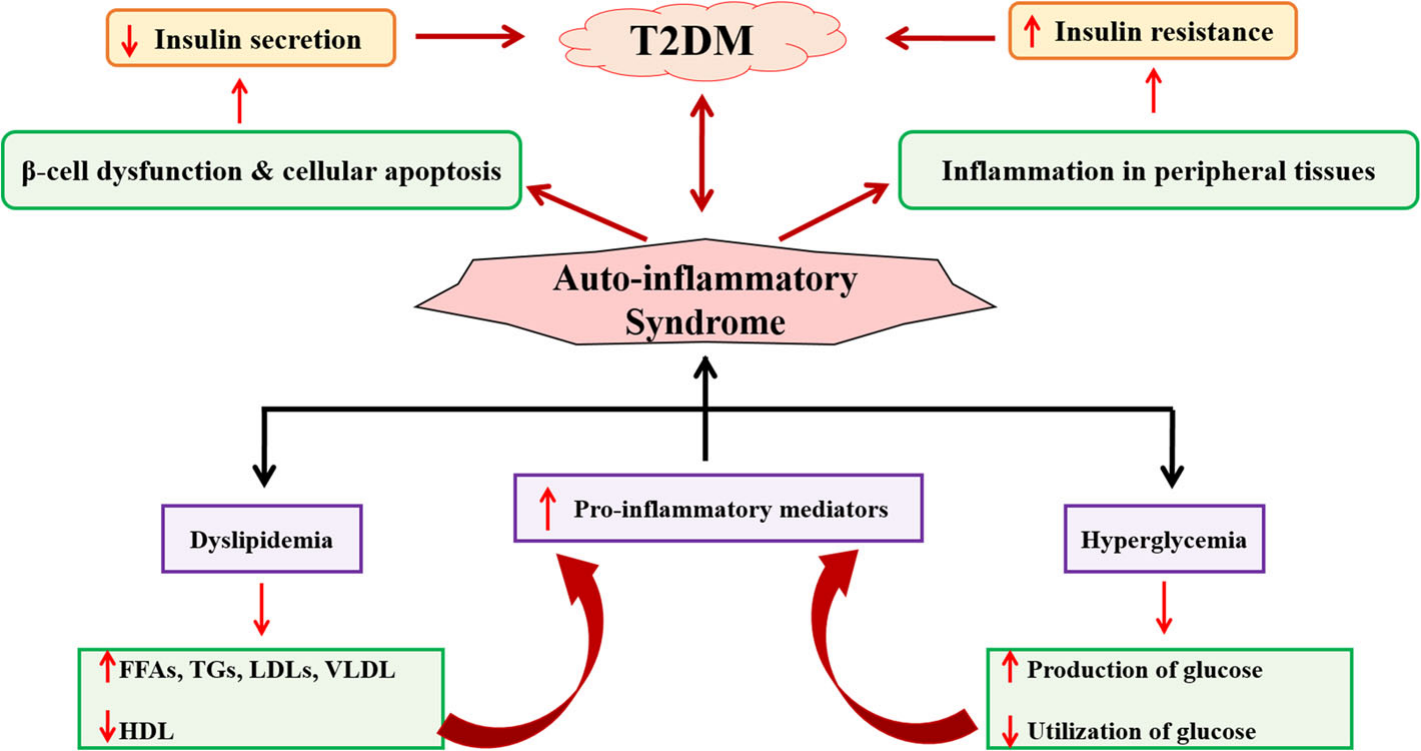
Mechanism of hyperglycemia- and dyslipidemia-induced inflammation for the development of IR and T2DM. Hyperglycemia and dyslipidemia collectively provoke the activation of pro-inflammatory mediators through the involvement of several metabolic pathways. Once, these pro-inflammatory mediators are released, they induce tissue-specific inflammation due to which IR in peripheral tissues and impaired insulin secretion in pancreatic islets occur that ultimately lead to overt T2DM. Adapted from Akash et al. [30]

Glucolipotoxicity is a general term which is collectively used for the combination of glucotoxicity and lipotoxicity. These two terms are collectively responsible to activate the release of various pro-inflammatory mediators which lead to the development of tissue-specific IR and impaired insulin secretion from β-cells of pancreatic islets (Fig. 13). Adipocytes are the main sites for the storage of fats and energy supplied to the body, is also regulated by the adipocytes. When accumulation of lipids exceeds the energy expenditure, then most of the excess amount is stored in the form of FFAs in adipose and other insulin-sensitive tissues. When fat storage and energy supply is impaired in adipose tissues, elevation of FFAs levels in plasma occurs which is converted into the triglycerides and stores in non-adipose tissues [148]. The ectopic storage of FFAs metabolites (mostly triglycerides) results in lipotoxic effects in peripheral tissues (Fig. 5). In addition to this, elevated levels of FFAs in plasma may also interfere with insulin signaling pathways notably IRS-1 serine phosphorylation in peripheral tissues via activation of PKC and inhibition of IKK and JNK [145]. Hence, it has been evidenced that glucolipotoxicity is one of the major contributor for the development of tissue-specific IR.

### Treatment strategies

Development of IR is one of the major hallmark for pathogenesis of T2DM. To control the propagation of IR is one of the most important targeted treatment. For the development of IR, several factors are involved (Fig. 1) and suppression of these causative factors can help decrease the incidences of IR development. Several treatment strategies have been used to overcome the development of IR. The most important ones have been described here in the following sub-sections.

### Interleukin-1 receptor antagonist

Interleukin-1 receptor antagonist (IL-1Ra) is naturally occurring anti-inflammatory cytokine of interleukin-1 family. It competitively binds with IL-1RI and prevent the binding of IL-1β and antagonizes its effects. It has been evidenced from several experimental studies that imbalance between IL-1Ra and IL-1β generates inflammation in various parts of the body where IL-1RI is present [4, 12]. Moreover, it has also been found that expression of IL-1Ra is strongly correlated with the development of IR, impaired insulin secretion and T2DM [4, 149]. Treatment of human recombinant IL-1Ra improves normoglycemia, insulin sensitivity in adipose and peripheral tissues, and insulin secretion from β-cells of pancreatic islets impairs [31, 150, 151]. This is one of the most important treatment strategy that anti-inflammatory agent might indeed prevent the development of IR and improves glycemia. One of the main shortcoming of IL-1Ra is its short biological half-life and to overcome this problem, high doses with frequent dosing intervals are required to achieve desired therapeutic effects. To overcome this problem, several treatment strategies have been applied to prolong the biological half-life and therapeutic effects of IL-1Ra [29].

### Salicylates

Salicylates are an important class of anti-inflammatory agents. They are used in variety of inflammatory diseases and syndromes. Inflammation plays a crucial role for the development of IR and T2DM, therefore, by using salicylates as an alternate treatment strategy, it has been found that salicylates can imporve insulin sensitivity via inhibition of NF-κB and IKKβ [82] and glucose tolerance [152, 153].

### Anti-TNF approaches

In the above sections, it has been briefly described that TNF-α is one of the most important pro-inflammatory mediator that is responsible to induce IR in adipocytes and peripheral tissues. Inhibition of TNF-α production might be one of the choice to prevent the development of IR and pathogenesis of T2DM [4]. Recently, infliximab has been demonstrated to improve insulin signaling and inflammation especially in the liver in rodent model of diet-induced IR [154]. Similarly, using anti-TNF-α antibodies also improve the insulin sensitivity in peripheral tissues [155]. Lo et al. demonstrated that etanercept therapy can also improve total concentration of adiponectin which is anti-inflammatory adipokine and improved insulin sensitivity [155]. Keeping in view the decisive role of TNF-α in pathogenesis of IR, several anti-TNF-α treatment strategies have been utilized to prevent the pathogeneis of IR and development of T2DM. TNF-null(^−^/^−^) mice significantly improved the glucose tolerance and insulin sensitivity [156]. Similarly, anti-TNF-α treatment has also shown to prevent the IR in Sprague–Dawley rats [157] while neutralization of TNF-α also prevented IR in hepatocytes [158]. Few controversial studies have also demonstrated that using TNF-α blockade has no effect on IR [159] which indicates that TNF-α blockade is not a treatment of choice as its production is dependent on the generation of IL-1β and activation of various transcriptional mediated pathways.

### Anti-chemokine approaches

It has been thought that chemokines activately participate in the development of IR by potentiating the inflammation in adipocytes. Moreover, genetic inactivation of these chemokine signaling [52, 53, 160] or inhibition of their axis [161, 162] by pharmacological approaches have been shown to improve the insulin sensitivity in adipocytes and peripheral tissues. In recent studies, it has been found that use of anti-chemokine antibodies and/or antagonists has shown to improve the insulin sensitivity [163, 164]. The results of these studies illustrate that inhibition and/or neutralization of chemokines may be considered as an alternate therapeutic tool for the treatment of IR and T2DM.

### Pharmaceutical chaperones

ER stress, as mentioned in the above sections, is a key link between IR and T2DM [165]. Blockade of ER stress is one of the treatment option to prevent the development of IR and pathogenesis of T2DM. In the recent years, various pharmaceutical chaperones, notably endogenous bile acids and the derivatives of these bile acids such as ursodeoxycholic acid (UDCA), 4-phenyl butyric acid (PBA) have been investigated that have proven to have the ability to modulate the normal functioning of ER and its folding capacity [28]. Ozcan et al. [166] used pharmaceutical chaperone (UDCA) to investigate its therapeutic effects on obese and diabetic mice. The results of this study indicated that UDCA significantly improved insulin sensitivity and normoglycemia.

### Thiazolidinediones

Low-grade local and/or systemic inflammation, as discussed above, plays its crucial role in the development of IR and pathogenesis of T2DM. Induction of low-grade inflammation activates several metabolic and/or transcriptional mediated pathways that are responsible to provoke the pathogenesis of IR. Thiazolidinediones also known as glitazones, are one of the most important insulin sensitisers. They are the agonists of peroxisome proliferator-activated receptors-gamma (PPARγ). It has been found that thaizolidinediones have the ability to improve insulin action and decrease IR [167, 168].

#### Expert opinion

Inflammatory responses play a crucial role in the pathogenesis and development of IR which is one of the main causative factor for the etiology of T2DM. Inflammatory responses are induced through the activation of various pro-inflammatory and oxidative stress mediators via involment of various transcriptional mediated pathways. To stop the inflammatory responses in IR development is one of the key treatment strategy. In this areticle, we have comprehensively highlighted the up-to-date scientific knowlesge of role of inflammatory responses in IR development and its treatment strategies.

## Conclusions

IR plays a crucial role for the pathogenesis and development of T2DM and its associated complicaitons. It has been evidenced that development of IR is strongly associated with various factors and the findings discussed here, strongly suggest that IR is closely interlinked with dysregulation of various metabolic and/or transcriptional mediated pathways, activation of various pro-inflammatory, oxidative and/or ER stress mediators in both experimental animal models and diabetic humans. Activations of various pro-inflammatory, oxidative and/or ER stress mediators and adipokines, and abnormal metabolism of glucose and lipids can lead to the development of tissue-specific IR. This intriguing notion that pro-inflammatory mediators, metabolic and transcriptional mediated pathways are decisively involved to provoke the pathogenesis of IR, has also been supported by many clinical observations where IR has been strongly correlated with systemic and/or local low-grade chronic inflammation. Based on the findings mentioned in above sections, anti-inflammatory treatment strategies are one of the best choice to prevent the the pathogenesis of IR, but the studies conducted to investigate the role of anti-inflammatory strategies for the prevention of IR are still in their beginning stages and need to be focused further in future studies for more better and improved clinical outcomes.

## Abbreviations

AMPK: AMP-activated protein kinase
CRP: C-reactive proteins
IKK: Inhibitor kB kinase
IKK-β: IκB kinase β
IL-1Ra: Interleukin-1 receptor antagonist
IL-1RI: Interleukin-1 receptor type I
IL-6: Interleukin-6
IR: Insulin resistance
LPS: Lipopolysaccharide
MCP-1: Monocyte chemotactic protein
NF-κB: Nuclear factor kappa-B cells
PKC: Protein kinase C
PPARγ: Peroxisome proliferators-activated receptor gamma
SOCS: Suppressor of cytokine signaling
STAT5B: Signal transducer and activator of transcription 5B
T2DM: Type 2 diabetes mellitus
TLRs: Toll like receptors
TNF-α: Tumor necrosis factor-alpha
